# Sparse Temporal AutoEncoder for ECG Anomaly Detection

**DOI:** 10.3390/s26051589

**Published:** 2026-03-03

**Authors:** Radia Daci, Abdelmalik Taleb-Ahmed, Luigi Patrono, Cosimo Distante

**Affiliations:** 1 Institute of Applied Sciences and Intelligent Systems, Consiglio Nazionale delle Ricerche, 73100 Lecce, Italy; radia.daci@isasi.cnr.it; 2 IEMN UMR CNRS 8520, Université Polytechnique Hauts-de-France, 59313 Valenciennes, France; 3 Department of Innovation Engineering, University of Salento, 73100 Lecce, Italy; luigi.patrono@unisalento.it

**Keywords:** electrocardiogram (ECG), unsupervised anomaly detection, Temporal Convolutional Network, sparse attention

## Abstract

Electrocardiogram (ECG) analysis is a fundamental tool for diagnosing various cardiac conditions; however, accurately distinguishing between normal and abnormal ECG signals remains challenging due to high inter-individual variability and the inherent complexity of ECG waveforms. In this study, We propose a novel Sparse Temporal Autoencoder (STAE) for unsupervised ECG anomaly detection that leverages Temporal Convolutional Networks (TCNs) to extract hierarchical features from both time-domain and frequency-domain representations of ECG signals. Unlike traditional approaches requiring annotated abnormal samples, the proposed model is trained exclusively on normal ECG data, making it well-suited for real-world deployment. A STAE integrates a masked signal reconstruction strategy and a hybrid sparse attention mechanism combining sparse block and sparse strided attention to capture critical temporal and spectral patterns efficiently. The proposed method is evaluated on the PTB-XL dataset, where it achieves the highest ROC-AUC of 0.872 among compared unsupervised methods while maintaining a low inference time of 0.009 s, demonstrating that STAE achieves state-of-the-art performance in ECG anomaly detection, highlighting its potential as a powerful tool for automated and intelligent ECG analysis.

## 1. Introduction

Cardiovascular diseases remain the leading cause of mortality worldwide, highlighting the importance of timely and accurate detection of cardiac abnormalities for effective diagnosis and treatment [1]. Electrocardiograms (ECGs) play a central role in monitoring cardiac activity and supporting clinical decision making [2]. However, manual interpretation of ECG signals is time-consuming, prone to human error, and affected by inter-observer variability [3].

To address these challenges, deep learning techniques have been widely adopted for ECG anomaly detection. Supervised learning methods, particularly convolutional neural networks (CNNs), have demonstrated strong performance when sufficient labeled data are available [4]. However, the reliance on large-scale annotated abnormal ECG datasets limits their applicability in real-world scenarios. Consequently, unsupervised and semi-supervised approaches have gained increasing attention. Representative methods include LSTM-based autoencoders for ECG heartbeat analysis [5], graph neural network-based models for multivariate time series [6], and generative approaches such as BeatGAN [7], DAGMM [8], ECGGAN [9], as well as recent advances such as Jiang et al. [10], MMAE-ECG [11], and MAAT [12].

Beyond purely time-domain analysis, time–frequency representations such as spectrograms have been shown to capture complementary spectral and rhythmic characteristics that are not easily observable in raw ECG signals. Methods leveraging time–frequency information, including TSRNet [13] and multi-scale fusion approaches [14], have reported improved anomaly detection performance. Despite these advances, existing methods often focus on either time-domain or time–frequency representations independently, or employ computationally expensive attention mechanisms, limiting their efficiency and scalability.

From a modeling perspective, effective ECG anomaly detection requires capturing both morphological and rhythmic variations across time and frequency domains, while remaining computationally efficient for long recordings. Existing approaches often address these aspects in isolation or rely on dense attention mechanisms that scale poorly. These limitations motivate a unified, efficient framework that jointly models temporal and spectral information under an unsupervised setting.

As a result, several practical challenges remain insufficiently addressed in existing unsupervised ECG anomaly detection methods: dependence on labeled abnormal data, limited exploitation of complementary time and frequency representations, and high computational cost when modeling long ECG sequences. By addressing these challenges, the proposed approach enables unsupervised ECG anomaly detection that (i) eliminates dependence on labeled abnormal data, (ii) jointly captures temporal and spectral characteristics within a unified framework, and (iii) maintains scalability to long ECG recordings through computationally efficient attention modeling. These considerations motivate the proposed Sparse Temporal Autoencoder (STAE).

Building on these motivations, we propose a Sparse Temporal Autoencoder (STAE) for unsupervised ECG anomaly detection. The proposed framework jointly models time-domain and time–frequency representations using a Temporal Convolutional Network (TCN)-based autoencoder enhanced with a hybrid sparse attention mechanism. By integrating sparse block and sparse strided attention, STAE effectively captures both local and long-range dependencies while maintaining computational efficiency.

The main contributions of this work are summarized as follows:

- We propose an efficient unsupervised ECG anomaly detection framework that learns exclusively from normal ECG recordings, addressing realistic clinical and monitoring scenarios where abnormal annotations are scarce or unavailable.
- We introduce a scalable representation learning strategy that jointly captures complementary time-domain and time–frequency ECG characteristics using dual TCN encoders, addressing limitations of existing approaches in which these representations are either processed independently or combined using computationally expensive architectures that do not scale well to long ECG sequences.
- We design a hybrid sparse attention mechanism tailored to long ECG sequences, which enables effective modeling of both local and long-range temporal dependencies while substantially reducing the computational cost compared to dense attention mechanisms.
- Extensive experiments on the PTB-XL dataset demonstrate that the proposed framework achieves state-of-the-art unsupervised anomaly detection performance with real-time inference capability, highlighting its suitability for large-scale and continuous ECG monitoring.

Importantly, the proposed design prioritizes computational efficiency and scalability, enabling real-time anomaly detection on long-duration ECG recordings without sacrificing detection accuracy.

The proposed STAE is evaluated on the PTB-XL dataset, where training is performed exclusively on normal ECG samples and testing includes both normal and abnormal signals to assess anomaly detection performance.

In this work, a normal ECG refers to recordings acquired from individuals without diagnosed cardiac abnormalities, as defined by the expert annotations provided in the PTB-XL dataset. These annotations are derived from clinical reports and cardiologist interpretations associated with each recording [15]. No clinical trials were conducted as part of this study; instead, the proposed method is evaluated using retrospective, publicly available ECG data, which is standard practice in the development and benchmarking of data-driven ECG anomaly detection methods [10,13].

## 2. Related Work

### 2.1. Unsupervised ECG Anomaly Detection

The detection of anomalies in ECG time-series signals is critical for the early diagnosis of cardiovascular diseases. Traditional automated ECG analysis methods are primarily based on supervised learning frameworks, which require large amounts of annotated data. For instance, Muñoz et al. [4] compared several deep learning architectures, including CNN, CNN–LSTM, and CNN–LSTM with attention, for the classification of cardiac conditions. While supervised methods have demonstrated strong performance, their dependence on labeled abnormal data limits their applicability in real-world clinical settings.

To address these limitations, numerous unsupervised anomaly detection approaches have been proposed for ECG and general time-series data. BeatGAN [7] employed an adversarial reconstruction framework to model normal patterns and identify anomalies based on reconstruction errors. DAGMM [8] combined deep autoencoders with Gaussian mixture models for joint representation learning and density estimation. Jiang et al. [10] proposed a dual-branch masked autoencoder to capture global rhythm and local morphological variations in ECG signals. More recently, MMAE-ECG [11] and MAAT [12] introduced multi-scale masking and attention-based strategies to improve unsupervised ECG anomaly detection.

Although these methods demonstrate strong detection capability, most unsupervised ECG approaches rely on either time-domain representations alone or computationally expensive architectures that limit scalability to long ECG recordings.

### 2.2. Time-Domain and Time–Frequency ECG Representations

Time-domain ECG analysis focuses on waveform morphology and temporal dynamics, including the shape and duration of the P, QRS, and T waves as well as beat-to-beat timing relationships [16]. These characteristics are essential for identifying morphological abnormalities and rhythm irregularities.

Time–frequency representations, such as spectrograms, provide a complementary view by characterizing how spectral energy evolves over time [17]. This perspective is particularly effective for capturing rhythm instability, frequency dispersion, and transient oscillatory patterns that may be less evident in raw time-series signals.

Several recent works have demonstrated the benefit of incorporating time–frequency information for ECG anomaly detection. TSRNet [13] combined temporal and spectral restoration to emphasize clinically relevant ECG deviations, while other multi-scale fusion approaches [14] reported improved robustness by integrating spectral features.

Despite these advances, existing methods typically process time-domain and time–frequency representations independently or combine them using dense architectures that do not scale efficiently to long ECG sequences.

### 2.3. TCN-Based Models for ECG Analysis

Temporal Convolutional Networks (TCNs) have been widely adopted for time-series modeling due to their ability to capture long-range temporal dependencies using causal and dilated convolutions. TCN-based autoencoders have shown strong performance in unsupervised anomaly detection for generic time-series data [18,19].

In the ECG domain, TCN-based architectures have primarily been explored for supervised tasks such as heart rate analysis and arrhythmia classification [20]. These models demonstrate strong temporal modeling capability but typically operate exclusively in the time domain.

To date, the use of TCNs for unsupervised ECG anomaly detection remains limited, and existing approaches do not explicitly integrate complementary time–frequency representations within a unified and scalable framework.

### 2.4. Attention Mechanisms for Long ECG Sequences

Attention mechanisms have been introduced in ECG deep learning models to emphasize informative temporal regions and improve representation learning. Recent approaches often rely on dense or low-rank attention formulations [21], which provide flexible context modeling but incur quadratic or near-quadratic computational complexity with respect to sequence length.

Such attention mechanisms become impractical for long ECG sequences and continuous monitoring scenarios, where computational efficiency and real-time inference are critical requirements.

In contrast to the above approaches, the proposed Sparse Temporal Autoencoder (STAE) integrates dual TCN encoders for time-domain and time–frequency representations within an unsupervised reconstruction framework, and introduces a hybrid sparse attention mechanism tailored for long ECG sequences. This design enables efficient modeling of both local and long-range dependencies while maintaining scalability and real-time inference capability.

## 3. Methodology

In this paper, we present STAE, our unsupervised anomaly detection method for ECG (electrocardiogram) data, which operates in both the time-domain and spectrogram (frequency-domain) representations. Our model is designed to reconstruct ECG signals using a combination of temporal encoding, spectrogram encoding, and decoding. This is achieved through a TCN (Temporal Convolutional Network) autoencoder enhanced with a sparse cross-attention mechanism.

### 3.1. Problem Description

Given an ECG signal $X\in R^{T}$, the objective of this work is to identify abnormal cardiac patterns using an unsupervised reconstruction-based approach. The proposed model is trained only on normal ECG signals to learn a mapping that reconstructs the input signal $\hat{X}$ and estimates an associated uncertainty σ. During inference, deviations between *X* and $\hat{X}$, weighted by σ, are used to compute an anomaly score *S*, where higher values indicate a higher likelihood of abnormality.

### 3.2. STAE Architecture

**Figure 1:**
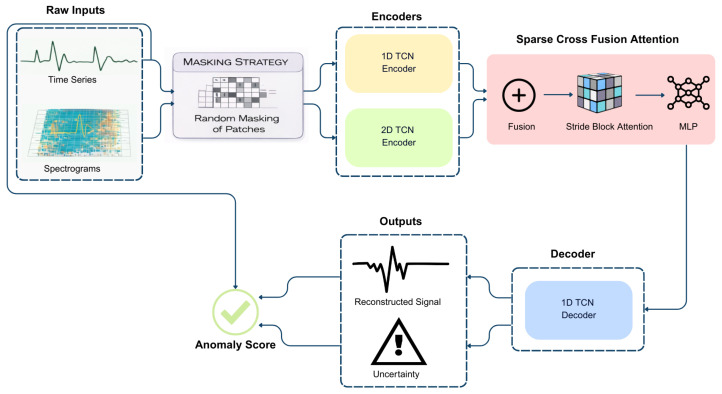
Overview of the proposed Sparse Temporal Autoencoder (STAE) architecture for unsupervised ECG anomaly detection. Raw ECG time-series signals and their corresponding time–frequency representations are first processed using a random patch-based masking strategy. Temporal and spectral features are extracted through TCN-based encoders and fused via a sparse cross-fusion module employing strided block attention. The refined latent representation is decoded to reconstruct the ECG signal and estimate uncertainty, which are jointly used to compute the anomaly score.

Our STAE model processes ECG signals in both the time-domain and spectrogram representations. The use of both time-domain and time–frequency representations is motivated by their complementary characteristics in ECG analysis. The time-domain signal preserves morphological information such as waveform shape and temporal dynamics, which are essential for identifying abnormal cardiac patterns. In contrast, the time–frequency representation highlights spectral and rhythmic variations that may not be easily observable in the raw signal. By jointly modeling these two representations, the proposed STAE captures a more comprehensive description of normal ECG behavior, improving its sensitivity to anomalous patterns. The model applies random patch-based masking to both domains. It utilizes a TCN-Encoder1D to extract features from time-series ECG data and a TCN-Encoder2D to capture spectrogram features. These extracted features are then integrated and refined through StridedBlockAttention, a cross-attention mechanism that enhances feature fusion between time and frequency domains. The transformed representation is further processed by a Multi-Layer Perceptron (MLP) before being reconstructed using a TCN-Decoder1D. Finally, the anomaly score is determined by comparing the reconstructed ECG signal and its associated variation with the original time-series data, as illustrated in Figure 1. This approach aligns with previous studies that have demonstrated the effectiveness of Temporal Convolutional Networks (TCNs) for ECG anomaly detection, such as the studies proposed in [20,22].

**Figure 2:**
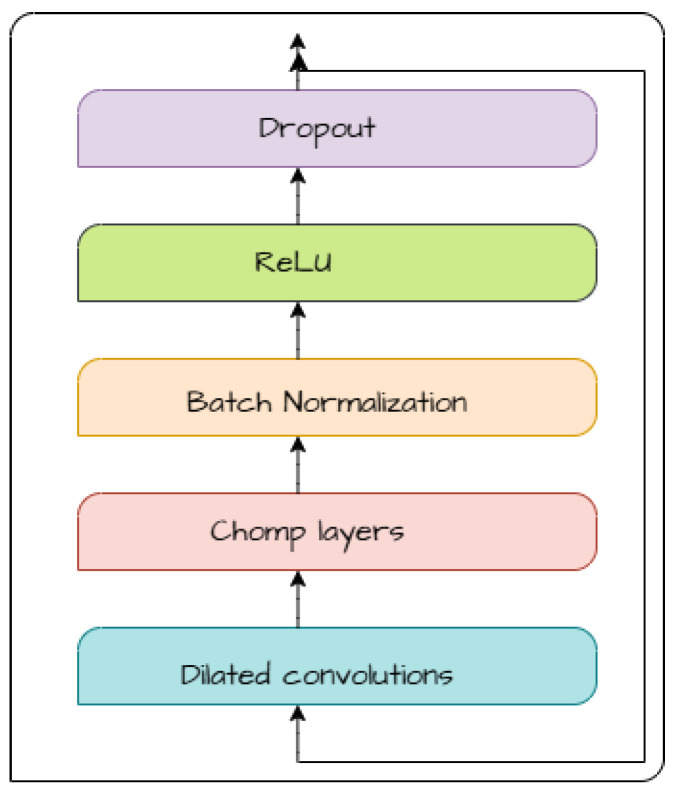
Structure of a Temporal Convolutional Network (TCN) block used in the encoder and decoder of the proposed STAE model. Each block consists of dilated causal convolutions followed by chomp layers to preserve temporal alignment, batch normalization, ReLU activation, dropout, and residual connections to enable stable and effective temporal feature learning.

1. Spectral signal and Masking-Out procedure:Generate a time–frequency representation from time series using the Short-Time Fourier Transform (STFT) and a masking strategy for both domains that partially obscures the input data. This approach, adapted from [13], encourages the model to focus on learning essential signal patterns from the training dataset.
2. TCN-based encoders: The STAE model integrates two TCN-based encoders, TCN-Encoder1D and TCN-Encoder2D, designed to extract temporal and spectral representations of ECG signals. These encoders ensure a comprehensive understanding of both time-series and frequency-domain features before fusion for anomaly detection. Both encoders employ Temporal Blocks as their fundamental building units, as illustrated in Figure 2, incorporating the following:
  Dilated convolutions to augment the receptive field, enabling the model to capture both local variations and long-range dependencies.
  Chomp layers to maintain temporal alignment and prevent unwanted shifts in feature extraction.
  Batch Normalization and Dropout to stabilize training and enhance generalization.
  ReLU activations for efficient non-linear feature transformation.
  Residual connections to preserve gradient flow and prevent vanishing gradients during deep feature extraction.
  Where TCN-Encoder1D consists of three stacked Temporal Blocks, producing a feature representation, and TCN-Encoder2D is composed of four stacked Temporal Block Outputs for spectral feature representation.
3. Sparse cross fusion attention: This process is enhanced through concatenation, sparse attention, and MLP transformations before being passed to the decoder for ECG reconstruction. After passing the input ECG data through both TCN 1D and 2D encoders, the extracted temporal features (from TCN-Encoder1D) and spectral features (from TCN-Encoder2D) are concatenated. This concatenation ensures that information in both the time domain and frequency domain is available for subsequent processing, allowing the model to capture a more comprehensive latent representation. Instead of directly feeding the concatenated features into the decoder, STAE applies a sparse attention mechanism [23], specifically Strided Block Attention, to refine feature interactions. Unlike traditional self-attention, which computes attention across the entire sequence, it suffers from high computational costs. Strided Block Attention divides the input sequence into blocks of a fixed size (block size) and applies attention only within each block using a stride-based mechanism rather than across the whole sequence, which limits the number of attention operations while still capturing essential local dependencies and incorporating global contextual information.

Concretely, within each block, only query positions sampled every *S* time steps participate in structured attention, and each such query attends exclusively to key positions sampled with the same stride inside the same block. All other query–key interactions are suppressed using a large negative mask value, enforcing a block-wise strided sparsity pattern. To further incorporate global contextual information at negligible cost, the masked attention logits are averaged across the query dimension, and this global mean-logit term is added back to the logits prior to softmax normalization.

This approach reduces computational overhead while still allowing information to flow across the entire sequence.

Computational Complexity Analysis: Standard self-attention mechanisms incur quadratic complexity $O(T^{2})$ with respect to the sequence length *T*, which becomes prohibitive for long ECG recordings. In the proposed strided block attention, the sequence is first partitioned into blocks of size *B*, and structured attention interactions are restricted to stride-sampled query and key positions within each block. As a result, each block contains approximately ${\left(B/S\right)}^{2}$ effective attention interactions, leading to an overall effective interaction complexity on the order of $O\;\left(T\cdot \frac{B}{S^{2}}\right)$.

In addition, a lightweight global context term is incorporated by averaging the masked attention logits across the query dimension, which introduces only linear overhead with respect to the sequence length. This design substantially reduces the number of meaningful attention interactions while preserving both local and global temporal dependencies, enabling efficient modeling of long ECG sequences. Algorithm 1 details the implementation of the proposed Strided Block Attention mechanism, including the construction of the strided block mask and the global mean-logit fusion strategy.
**Algorithm 1** Strided Block Attention with Global Mean-Logit Fusion.**Require:** $Q_{in},K_{in},V_{in}\in R^{N\times T\times D}$, heads *H*, block size *B*, stride *S***Ensure:** $Y\in R^{N\times T\times D}$  1:d←D/H  2:$Q\leftarrow \mathrm{reshape}\_\mathrm{heads}\left(W_{Q}Q_{in}\right)\in R^{N\times H\times T\times d}$  3:$K\leftarrow \mathrm{reshape}\_\mathrm{heads}\left(W_{K}K_{in}\right)\in R^{N\times H\times T\times d}$  4:$V\leftarrow \mathrm{reshape}\_\mathrm{heads}\left(W_{V}V_{in}\right)\in R^{N\times H\times T\times d}$  5:$M\leftarrow 0_{T\times T}$                                                                                       ▹ Strided-block mask  6:$n_{b}\leftarrow \left\lceil T/B\right\rceil$  7:**for** b←0 **to** $n_{b}-1$ **do**  8:       s←b·B  9:       e←min(s+B,T)10:       **for** i←s **to** e−1 **step** *S* **do**11:             **for** j←s **to** e−1 **step** *S* **do**12:                   M[i,j]←113:             **end for**14:       **end for**15:**end for**16:$A\leftarrow \left(QK^{⊤}\right)/\sqrt{d}$                                                                                        ▹$A\in R^{N\times H\times T\times T}$17:$A\leftarrow A+\left(-10^{9}\right)\cdot \left(1-M\right)$                                                              ▹ Mask invalid pairs18:G←mean(A,dim=query)                                                                    ▹$G\in R^{N\times H\times 1\times T}$19:$\tilde{A}\leftarrow A+G$                                                                          ▹ Global mean-logit fusion20:$P\leftarrow \mathrm{softmax}(\tilde{A},\;\dim=\mathrm{key})$21:P←Dropout(P)22:Z←PV                                                                                                    ▹$Z\in R^{N\times H\times T\times d}$23:$Y\leftarrow W_{O}\;\mathrm{concat}\_\mathrm{heads}\left(Z\right)$24:**return** *Y*

After feature refinement using sparse attention, the transformed representation is passed through a Multi-Layer Perceptron (MLP) that enhances feature projection and prepares the representation for decoding.

4. TCN-Decoder1D: In the STAE model, it is responsible for reconstructing the original ECG signal along with its variation (uncertainty estimate), which is later utilized in the anomaly score computation. It employs Temporal Blocks as its core building components, incorporating Dilated Convolutions, Chomp Layers, Residual Connections, and ReLU Activation, as shown in Figure 2, while omitting Batch Normalization and Dropout.

### 3.3. Training Objective

The proposed STAE model is trained using an uncertainty-aware reconstruction objective. Given an input ECG signal $X\in R^{T}$, the model predicts a reconstructed signal $\hat{X}$ along with a corresponding uncertainty estimate σ, where σ reflects the model’s confidence in its reconstruction.

The training loss is defined as

(1) $$L=E\left[\exp\left(-\sigma \right)\cdot {\left(\hat{X}-X\right)}^{2}+\sigma \right],$$

where the exponential weighting encourages accurate reconstruction while the uncertainty regularization term prevents trivial solutions.

During training, random patch-based masking is applied to both the time-domain and time–frequency representations, forcing the model to recover missing signal segments from contextual information. This self-restoration strategy promotes learning robust temporal and spectral patterns of normal ECG signals and improves generalization to unseen abnormal signals.

### 3.4. Anomaly Score

To assess the abnormality of an electrocardiogram (ECG) signal, we employ a reconstruction-based anomaly detection approach based on the outputs $\hat{X}$ and σ produced by the trained model. The squared reconstruction error is computed as

(2) $$E={\left(\hat{X}-X\right)}^{2}$$

To account for varying confidence levels across different regions of the ECG signal, we apply an uncertainty-aware weighting mechanism [13]. Specifically, the reconstruction error is scaled by the inverse of the predicted variance:

(3) $$S=\left[\exp\left(-\sigma \right)\cdot {\left(\hat{X}-X\right)}^{2}+\epsilon \right],$$

where $\epsilon =1\times 10^{-10}$ is a small constant added to ensure numerical stability.

The value of ϵ is deliberately chosen to be sufficiently small so that it does not affect the magnitude of the anomaly score, while preventing potential numerical issues such as division-by-zero or undefined gradients when the reconstruction error approaches zero. This choice is consistent with common practice in reconstruction-based anomaly detection models, where a small constant is introduced solely to ensure numerical stability during optimization and inference [24].

For each ECG sample, the anomaly score is computed as the mean over all time points:

(4) $$S_{\mathrm{sample}}=\frac{1}{T}\sum_{t=1}^{T}S_{t}$$

To obtain a normalized anomaly score, min–max normalization is applied [25]:

(5) $$S=\frac{S_{\mathrm{sample}}-\min\left(S_{\mathrm{sample}}\right)}{\max\left(S_{\mathrm{sample}}\right)-\min\left(S_{\mathrm{sample}}\right)},$$

where S∈[0,1], and higher values indicate a higher likelihood of abnormality.

Note that the anomaly score differs from the training objective by excluding the uncertainty regularization term, as the score is used solely to quantify abnormality during inference rather than to optimize model parameters.

## 4. Experiments

### 4.1. ECG Dataset and Preprocessing

We utilize the PTB-XL dataset [15] for our experiments. PTB-XL is a large-scale, publicly available ECG dataset that contains 12-lead electrocardiogram recordings sampled at 500 Hz, with each recording spanning 10 s per patient. The dataset includes diverse clinically annotated cardiac conditions, making it well-suited for evaluating unsupervised ECG anomaly detection methods.

To ensure fair comparison with recent state-of-the-art approaches, we follow the official data partitioning strategy adopted in [13]. Specifically, the training set consists of 8167 ECG recordings labeled as normal, while the test set contains 912 normal samples and 1248 abnormal samples. In accordance with the unsupervised learning setting, only normal ECG recordings are used during training, whereas both normal and abnormal signals are included during testing.

Prior to model training and evaluation, a standardized ECG preprocessing pipeline is applied to improve signal quality and ensure reproducibility. All experiments use the high-resolution PTB-XL recordings sampled at 500 Hz, and all 12 ECG leads are retained without lead selection. For each lead, the raw ECG signal is denoised using a sequence of digital filters implemented with the HeartPy library. Specifically, a high-pass filter with a cutoff frequency of 1 Hz is applied to remove baseline wander, followed by a notch filter to suppress power-line interference, and a low-pass filter with a cutoff frequency of 25 Hz to attenuate high-frequency noise. This filtering strategy follows common practice in ECG signal analysis and has been widely adopted in prior studies on the PTB-XL dataset.

After denoising, each ECG recording is independently normalized per lead using min–max scaling to map signal amplitudes into the range [−1,1]. To further ensure data quality, ECG recordings that cannot be successfully processed by the HeartPy signal quality check are excluded from both the training and testing sets. This preprocessing pipeline is applied consistently across all experiments to guarantee reliable and reproducible performance evaluation [10].

### 4.2. Evaluation Metrics

We assess the network’s performance using several evaluation metrics. counting AUC-ROC(AUC), Precision, Accuracy, Recall, and F1-score. Additionally, we measure the network’s complexity based on the number of trainable parameters and inference time (in seconds). The definitions of these evaluation metrics [26] are as follows:

AUC-ROC(AUC): represents the area under the Receiver Operating Characteristic curve and evaluates the discrimination capability of the model across different decision thresholds.

Accuracy: represents the proportion of accurately classified instances over the total number of samples [27].

(6) $$\mathrm{Accuracy}=\frac{TP+TN}{TP+TN+FP+FN}$$

Precision: measures the proportion of correctly predicted positive cases out of all predicted positive cases.

(7) $$\mathrm{Precision}=\frac{TP}{TP+FP}$$

Recall: quantifies the proportion of actual positives that were correctly identified.

(8) $$\mathrm{Recall}=\frac{TP}{TP+FN}$$

F1-score: a harmonic mean that balances the precision and recall of the two measurements.

(9) $$F1=2\times \frac{\mathrm{Precision}\times \mathrm{Recall}}{\mathrm{Precision}+\mathrm{Recall}}$$

where

TP (True Positives): Number of true positive cases.

TN (True Negatives): Number of true negative cases.

FP (False Positives): Number of false positive cases.

FN (False Negatives): Number of false negative cases.

The optimal anomaly detection threshold is determined using Youden’s Index, a widely adopted criterion in biomedical signal analysis for balancing sensitivity and specificity. Given the predicted anomaly scores and ground-truth labels, the Receiver Operating Characteristic (ROC) curve is first constructed by evaluating the true positive rate (TPR) and false positive rate (FPR) across a range of decision thresholds. The true negative rate (TNR) is then computed as TNR=1−FPR.

Youden’s Index is defined as J=TPR+TNR−1, and the optimal threshold is selected as the value that maximizes *J* over the ROC curve. This criterion identifies the operating point that provides the best trade-off between correctly detecting abnormal ECG signals and avoiding false alarms. The adopted threshold selection strategy follows established practice in ECG analysis and medical decision support systems, consistent with the methodology described in [28].

### 4.3. Implementation Details

Our model is developed using the PyTorch 2.6.0 library and trained on a single NVIDIA TITAN RTX GPU with Total Memory(GB): 24. The model is trained for 50 epochs with a batch size of 32 using the AdamW optimizer [29] with an initial learning rate of 5 × 10^−5^, a weight decay of 1 × 10^−4^.

### 4.4. Performance Comparison

We evaluate the performance of our STAE against multiple state-of-the-art unsupervised methods for time-series anomaly detection, including Zheng et al. [30], BeatGAN [7], Jiang et al. [10], MMAE-ECG [11], and the unsupervised TSRNet [13], which leverages both time-series and time–frequency representations. A quantitative comparison with these methods is reported in Table 1. In addition Table 2 summarizes the classification performance of STAE alongside other unsupervised ECG anomaly detection methods, including Accuracy, Precision, Recall, and F1-score.

**Figure 3:**
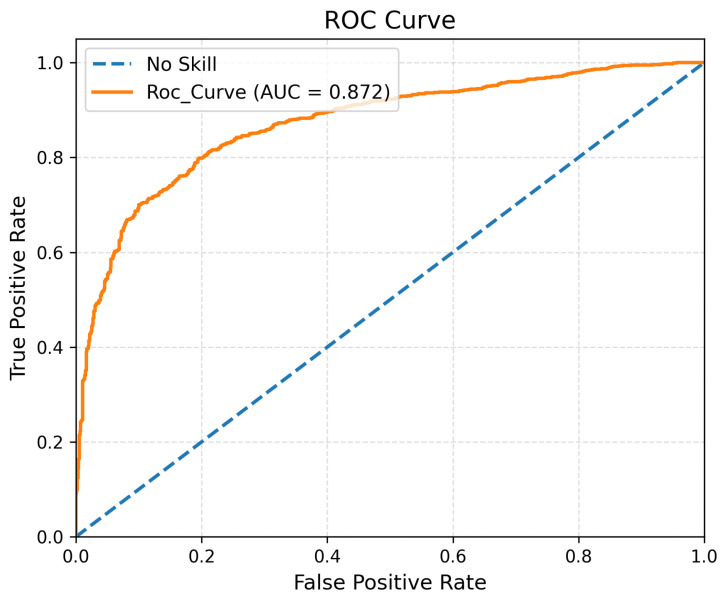
Receiver Operating Characteristic (ROC) curve of the proposed STAE model on the PTB-XL dataset. The dashed diagonal line represents the no-skill classifier, while the solid curve corresponds to STAE, achieving an AUC of 0.872.

**Figure 4:**
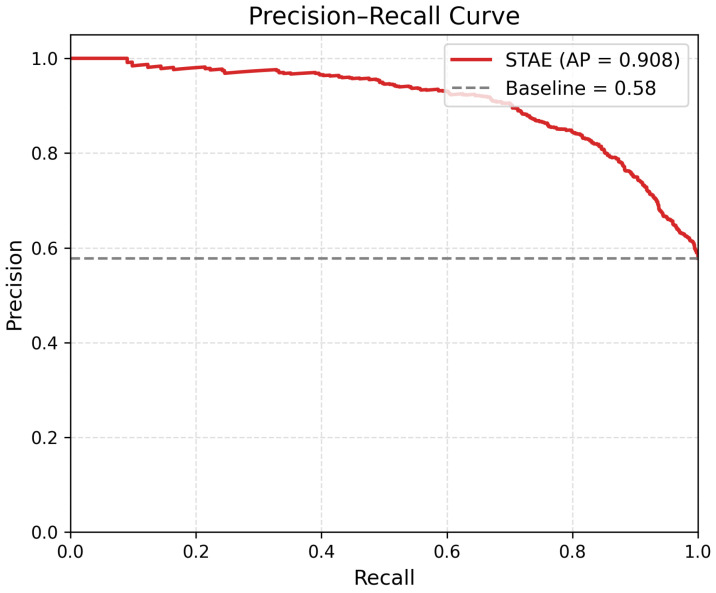
Precision–Recall (PR) curve of the proposed STAE model on the PTB-XL dataset. The dashed line denotes the baseline performance, while the proposed method achieves an average precision (AP) of 0.908.

**Figure 5:**
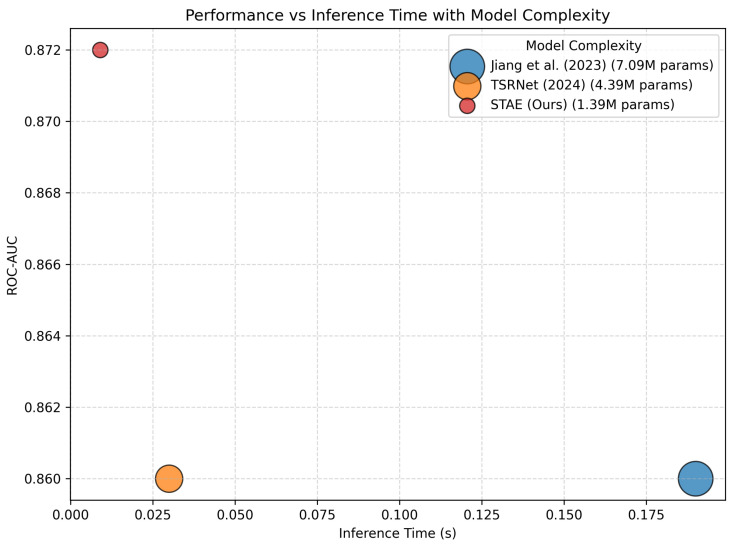
Comparison of detection performance (ROC-AUC) versus inference time for representative unsupervised ECG anomaly detection methods. Each point corresponds to a method. Bubble size represents model complexity in terms of the number of trainable parameters, where smaller bubbles indicate more compact models with fewer parameters [10,13].

Figure 3 and Figure 4 illustrate the ROC and Precision–Recall curves of the proposed STAE model, confirming its strong discriminative capability across different decision thresholds, while Figure 5 provides a visual comparison of detection performance, inference time, and model complexity.

Table 1 presents a comparative evaluation of the proposed STAE against representative state-of-the-art unsupervised ECG anomaly detection methods on the PTB-XL dataset. The comparison focuses on three complementary aspects: detection accuracy (ROC-AUC), model complexity (number of trainable parameters), and computational efficiency (inference time).

From an accuracy perspective, STAE achieves the highest AUC (0.872), outperforming existing unsupervised approaches, including models that rely on adversarial learning, multi-scale autoencoding, or time–frequency fusion. This indicates that the proposed joint temporal and spectral modeling, combined with sparse attention, leads to more effective characterization of normal ECG patterns and improved anomaly discrimination.

In terms of computational cost, STAE maintains a lightweight architecture with 1.39 M parameters, which is significantly smaller than several competing methods and only moderately larger than the most compact model (MMAE-ECG). Importantly, STAE achieves the fastest inference time (0.009 s), demonstrating that the improved detection performance is obtained without sacrificing real-time applicability.

Overall, Table 1 confirms that STAE provides a favorable trade-off between detection accuracy, model complexity, and inference efficiency. This balance validates the effectiveness of the proposed design and highlights its suitability for real-world ECG monitoring scenarios where both reliability and computational cost are critical.

While Table 1 evaluates detection performance (AUC), model complexity (number of parameters), and computational efficiency (inference time), Table 2 provides a complementary analysis based on threshold-dependent classification metrics, including Accuracy, Precision, Recall, and F1-score. Together, these evaluations offer a comprehensive assessment of both discriminative capability and practical detection behavior.

As reported in Table 2, STAE achieves the highest results across all classification metrics, with an Accuracy of 0.801, a Precision of 0.848, a Recall of 0.798, and an F1-score of 0.822. Compared with [10,13], the proposed method improves overall classification consistency while maintaining a balanced trade-off between precision and recall.

The relatively high precision indicates reliable identification of abnormal ECG segments with limited false positives, whereas the improved recall reflects effective detection of anomalous cases. The F1-score confirms that these gains are balanced rather than driven by emphasizing a single metric.

The consistent improvements observed across both detection-level evaluation (Table 1) and classification-level metrics (Table 2) suggest that the proposed joint temporal and time–frequency modeling with sparse attention enhances anomaly discrimination while preserving stable generalization performance.

Figure 5 provides a visual comparison of detection performance, inference time, and model complexity, where higher ROC-AUC values indicate better detection performance, lower inference time reflects faster execution, and smaller bubble sizes correspond to models with fewer trainable parameters.

The figure further illustrates the trade-off between detection accuracy and computational efficiency among representative unsupervised ECG anomaly detection methods. Models with larger complexity, such as Jiang et al. [10], achieve competitive ROC-AUC values but incur higher inference latency, while TSRNet [13] reduces inference time at the cost of increased model size.

In contrast, STAE lies in the upper-left region of the plot, indicating a favorable balance between high detection performance, low inference time, and reduced model complexity. This demonstrates that STAE achieves superior accuracy without increasing computational cost, making it well-suited for real-time and resource-constrained ECG monitoring applications.

### 4.5. Ablation Study on Sparse Attention Mechanisms

**Figure 6:**
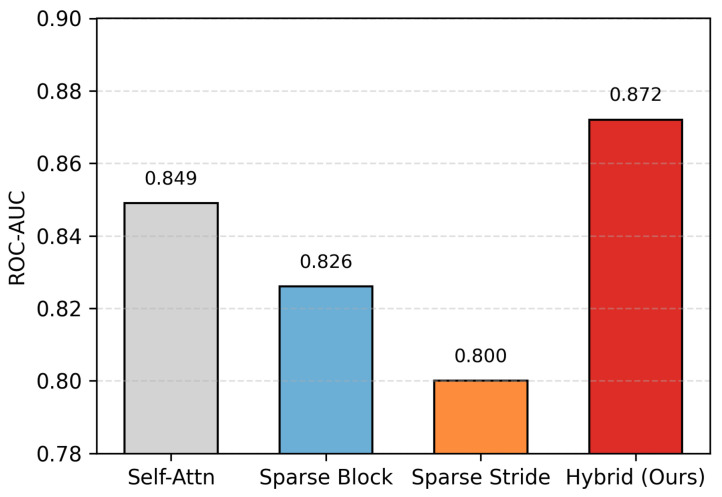
Ablation study on different attention mechanisms. The proposed hybrid sparse attention achieves the highest ROC-AUC, demonstrating the benefit of combining block and strided attention for ECG anomaly detection.

In this ablation study, we investigate the contribution of different attention mechanisms to the overall performance of the proposed STAE model. All experiments are conducted under identical settings, where only the attention module is varied while keeping all other components unchanged. Table 3 and Figure 6 summarize the comparison between standard self-attention, sparse block attention, sparse strided attention, and the proposed hybrid sparse attention.

Standard self-attention achieves an AUC of 0.849, while sparse block attention and sparse strided attention obtain lower AUC scores of 0.826 and 0.800, respectively. In contrast, the proposed hybrid sparse attention mechanism, which combines sparse block and sparse strided attention, achieves the highest AUC of 0.872. This improvement indicates that jointly modeling local dependencies and long-range interactions through the proposed hybrid sparse attention is more effective than using either sparse strategy alone, leading to enhanced anomaly detection performance.

From a modeling perspective, the superior performance of the hybrid sparse attention can be attributed to its ability to jointly capture fine-grained local temporal patterns and broader contextual dependencies within ECG signals. Sparse block attention emphasizes localized waveform structures, while sparse strided attention facilitates information exchange across distant temporal regions. By integrating these two complementary mechanisms, the proposed hybrid design enables more expressive feature interactions without incurring the full computational cost of dense self-attention. This result confirms that the performance gain arises from the attention design itself rather than increased model capacity, further validating the effectiveness of the proposed sparse attention strategy.

### 4.6. Ablation Study on the Time–Frequency Representation

To assess the contribution of the time–frequency representation in the proposed STAE framework, we conduct an ablation study comparing a time-domain-only configuration with the full model incorporating the spectrogram-based branch (Table 4). This analysis evaluates whether the additional 2D spectral encoder provides a meaningful performance gain beyond temporal modeling alone.

In the time-domain-only configuration, the model relies exclusively on the TCN-Encoder1D, with the spectrogram generation and TCN-Encoder2D removed, while all other components and training settings remain unchanged. This variant serves as a purely temporal baseline.

The full STAE model jointly processes the ECG signal in the time domain and its corresponding time–frequency representation obtained via Short-Time Fourier Transform (STFT), with temporal and spectral features fused through the proposed sparse cross-attention mechanism.

The results show that temporal modeling alone achieves an AUC of 0.781 with extremely low latency, indicating that TCN-based reconstruction can capture meaningful ECG patterns. However, incorporating the time–frequency branch leads to a substantial improvement, increasing the AUC to 0.872 while maintaining a low inference time of 0.009 s.

This performance gain demonstrates that spectral information provides complementary, non-redundant cues that are not fully captured by temporal modeling alone. In particular, certain abnormal cardiac patterns, such as rhythm irregularities and frequency-dependent disturbances, are more distinctly represented in the spectral domain. Importantly, the additional computational overhead remains modest and preserves real-time inference capability.

Overall, this ablation study confirms that the inclusion of the time–frequency branch significantly enhances anomaly detection performance while maintaining computational efficiency, thereby justifying its integration within the STAE framework.

## 5. Discussion

Clinical interpretation of reconstruction-based anomaly detection. While the proposed STAE framework is evaluated primarily using standard engineering metrics such as ROC-AUC and inference time, it is important to discuss the clinical relevance and interpretability of the detected anomalies [31]. In this work, STAE is designed as an unsupervised anomaly detection model trained exclusively on normal ECG signals, with anomalies identified through elevated reconstruction errors. Consequently, the model does not aim to classify specific cardiac pathologies but rather to detect deviations from learned normal ECG patterns.

Sensitivity to physiologically meaningful ECG deviations. From a signal interpretation perspective, the model is particularly sensitive to ECG abnormalities that introduce structural or rhythmic deviations, such as irregular beat intervals, altered waveform morphology, or changes in frequency content. These include rhythm disturbances (e.g., irregular RR intervals), abnormal QRS complexes, and waveform distortions that affect the temporal consistency or spectral distribution of the signal. Such deviations lead to increased reconstruction errors, as they differ from the normal patterns learned during training [32].

Role of time–frequency representation. The incorporation of a time–frequency representation further enhances sensitivity to abnormalities that manifest as changes in spectral energy distribution or periodicity, such as rhythm instability or subtle oscillatory disturbances. While the temporal branch captures morphological and timing-related variations, the spectral branch highlights frequency-domain inconsistencies that may not be clearly observable in the raw time series alone. This complementary behavior explains the improved anomaly detection performance observed in the ablation study.

Intended clinical role and the scope and limitations of the method. Reconstruction error is used as a generic anomaly score rather than a clinically interpretable diagnostic marker. Accordingly, STAE is not intended to replace clinical diagnosis or assign explicit pathological labels, but to serve as a decision-support and screening tool that flags potentially abnormal ECG segments for further expert review. The method adopts a data-driven definition of normality, operates at the segment level without localizing individual waveform components, and employs a fixed sparse attention pattern as a deliberate trade-off between modeling flexibility and computational efficiency, enabling scalable analysis of long-duration ECG recordings.

Dataset selection and benchmarking protocol. The experimental evaluation is conducted on the PTB-XL dataset, which is the largest publicly available and clinically diverse ECG dataset, comprising recordings from a broad population with substantial inter-individual variability, multiple diagnostic categories, and standardized acquisition protocols. PTB-XL is widely adopted as a benchmark for ECG representation learning and unsupervised anomaly detection. To ensure fair and consistent comparison, we follow experimental protocols used by recent unsupervised ECG anomaly detection methods [11,13], which also report results exclusively on PTB-XL, thereby avoiding confounding factors introduced by heterogeneous annotation standards or device-specific acquisition conditions.

Positioning of the contribution. From a methodological perspective, the contribution of STAE lies not in proposing a fundamentally new building block, but in the principled integration of mature components into a scalable and practically deployable unsupervised framework tailored to long ECG sequences.

Summary. Overall, STAE provides an efficient and robust anomaly detection mechanism that captures meaningful deviations in ECG structure and dynamics. By emphasizing unsupervised learning, computational efficiency, and scalability, the proposed framework offers practical value as a front-end screening solution for continuous ECG monitoring and large-scale cardiovascular data analysis.

## 6. Conclusions

In this study, we addressed the challenge of ECG anomaly detection, a task made difficult by the complex morphology of ECG signals and high inter-individual variability. To this end, we proposed STAE (Sparse Temporal Autoencoder), an unsupervised framework that combines masked signal reconstruction with a hybrid sparse attention mechanism to jointly model temporal and spectral dependencies in ECG signals.

Evaluated on the PTB-XL dataset, STAE achieved a ROC-AUC of 0.872, outperforming existing unsupervised approaches while maintaining extremely low inference latency (0.009 s) and a compact model size (1.39 M parameters). These characteristics make the proposed method particularly well-suited for real-time ECG monitoring and deployment in resource-constrained and large-scale clinical environments.

By integrating dual TCN-based encoders with a computationally efficient sparse attention mechanism, STAE provides a scalable and practical solution for unsupervised ECG anomaly detection in long-duration recordings. The proposed design effectively balances detection accuracy, computational efficiency, and modeling capacity, making it suitable for continuous monitoring and screening applications.

Future research may explore extending the proposed framework to additional ECG datasets and acquisition settings, as well as incorporating clinically interpretable mechanisms to further relate detected anomalies to specific waveform characteristics. Extending the approach to other physiological time-series signals also represents a promising direction for broader applicability.

## Figures and Tables

**Table 1:** Comparison of unsupervised ECG anomaly detection on the PTB-XL dataset. The best and second-best results are indicated using **boldface** and underlining, respectively.

Method	AUC	Params (M)	Inference Time (s)
Zheng et al (2022) [30]	0.757	-	-
BeatGAN (2022) [7]	0.799	-	-
Jiang et al (2023) [10]	0.860	7.09	0.19
TSRNet (2024) [13]	0.860	4.39	0.03
MMAE-ECG (2025) [11]	0.860	**0.398**	-
**STAE (Ours)**	**0.872**	1.39	**0.009**

**Table 2:** Comparison of unsupervised ECG anomaly detection methods on the PTB-XL dataset in terms of classification metrics. Accuracy, Precision, Recall, and F1-score. The best and second-best results are indicated using **boldface** and underlining, respectively.

Method	Accuracy	Precision	Recall	F1-Score
Jiang et al (2023) [10]	0.759	0.838	0.723	0.776
TSRNet (2024) [13]	0.780	0.841	0.764	0.801
**STAE (Ours)**	**0.801**	**0.848**	**0.798**	**0.822**

**Table 3:** Comparative analysis of different attention mechanisms based on Area Under the Curve (AUC) scores. The highest score is shown in **bold**.

Attention	AUC
Standard self-attention	0.849
Sparse block attention	0.826
Sparse strided attention	0.800
**Sparse strided block attention (Ours)**	**0.872**

**Table 4:** Ablation study on the contribution of the time–frequency branch.

Model Variant	AUC	Inference Time (s)
Time-domain only STAE	0.781	**0.003**
**Full STAE (Ours)**	**0.872**	0.009

## Data Availability

The experimental analysis in this study relies on the PTB-XL electrocardiogram (ECG) dataset, introduced by Wagner et al. [15]. The dataset is publicly available at: https://www.nature.com/articles/s41597-020-0495-6 (accessed on 15 December 2024). The model was implemented using PyTorch 2.6.0 (https://pytorch.org, accessed on 10 February 2025) and trained on a single NVIDIA TITAN RTX GPU (NVIDIA Corporation, Santa Clara, CA, USA; 24 GB memory). The source code developed for this study is publicly available at: https://github.com/ECGAI-Research/STAE (accessed on 6 January 2026).
